# Atherogenic index of plasma is associated with poor prognosis in diabetic patients with acute kidney injury

**DOI:** 10.3389/fendo.2026.1796454

**Published:** 2026-04-16

**Authors:** Manqiu Mo, Ling Pan

**Affiliations:** 1 Geriatric Department of Endocrinology, The First Affiliated Hospital of Guangxi Medical University, Nanning, China; 2 Department of Nephrology, The First Affiliated Hospital of Guangxi Medical University, Nanning, China

**Keywords:** acute kidney injury, all-cause death, diabetes, atherogenic index of plasma, prognosis

## Abstract

**Objective:**

To explore the correlation between atherogenic index of plasma (AIP) and all-cause death and renal non-recovery in diabetic patients with acute kidney injury (AKI).

**Methods:**

A retrospective analysis was conducted on the baseline and follow-up clinical data of diabetic patients with AKI in the First Affiliated Hospital of Guangxi Medical University from April 2010 to April 2025. The baseline AIP is calculated as log10 [TG (mg/dL)/HDL-C(mg/dL)]. Diabetic patients with AKI were divided into Q1, Q2, Q3, and Q4 groups based on the quartiles of baseline AIP. The differences in clinical data among different AIP groups were compared. The predictive value of AIP for all-cause death and non-recovery of renal function in diabetic patients with AKI was evaluated by drawing receiver operating characteristic (ROC) curves. The association between AIP and clinical outcomes in diabetic patients with AKI was analyzed using multivariable Cox regression and restricted cubic spline (RCS) regression.

**Result:**

A total of 1046 diabetic patients with AKI were enrolled, with a male to female ratio of 2:1. The average age was 62.40 ± 13.57 years. The average follow-up period was 101.13 ± 64.81 days. The all-cause mortality was 16.8%, and the rate of lack of renal recovery was 69.4%. The area under ROC curves for AIP in predicting all-cause death and lack of renal recovery in diabetic AKI patients were 0.805 and 0.782, respectively. The multivariate Cox and RCS regression analysis showed that after adjusting for confounding factors, the AIP level were non-linear associated with risk of all-cause death (HR = 5.427, 95%CI=2.999-9.821, inverted U-shape) and non-recovery of renal function (HR = 2.769, 95% CI = 2.085-3.677, S-shape) in diabetic patients with AKI. However, competing risk analysis revealed that after accounting for death as a competing event, AIP was no longer significantly associated with renal non-recovery (HR = 1.126, 95% CI: 0.864–1.467, P = 0.380).

**Conclusion:**

AIP is non-linearly associated with all-cause death in diabetic patients with AKI, and may serve as a useful biomarker for mortality risk stratification rather than for predicting renal recovery.

## Background

With rapid economic development and lifestyle changes, diabetes has become a serious global public health problem. According to the 11th edition of the International Diabetes Federation Diabetes Atlas, there were 589 million adult diabetes patients aged 20–79 worldwide in 2024. It is projected that this number will rise to 853 million by 2050. In 2024, approximately 3.4 million adults worldwide died from diabetes or its complications, accounting for 9.3% of all deaths (1).

In recent years, acute kidney injury (AKI) has been regarded as one of the complications of diabetes (2, 3). In addition, diabetes is also an independent risk factor for the onset and progression of AKI (4, 5). A cohort study showed that the incidence of AKI among diabetic patients was as high as 48.6%, significantly higher than that among non-diabetic patients (17.2%) (6). Compared with non-diabetic patients, the risk of death or lack of renal recovery in diabetic patients with AKI also increases (7, 8). If not identified early and treated promptly, diabetic patients with AKI are prone to progress to chronic kidney disease (CKD) or end-stage renal disease (ESRD), and may experience adverse clinical outcomes such as cardiovascular events, severe infections, multiple organ dysfunction syndrome (MODS), and death (9, 10).

Atherosclerosis is a major contributor to cardiovascular-related mortality in patients with diabetes (11, 12). Patients with diabetes typically exhibit atherosclerotic dyslipidemia, characterized by elevated triglycerides (TG), decreased high-density lipoprotein cholesterol (HDL-C) (13, 14). An animal experiment has demonstrated that AKI also further aggravates the inflammatory response at the site of atherosclerosis in mice, promoting the development of atherosclerosis (15). The atherogenic index of plasma (AIP) is a comprehensive lipid metabolism indicator calculated based on the ratio of TG to HDL-C, which can sensitively reflect the risk of atherosclerosis. High level of AIP is directly related to coronary heart disease (CHD), stroke, metabolic syndrome, and diabetic complications (16–19). However, to date, the relationship between AIP levels and prognosis in diabetic patients with AKI is still unclear. Therefore, in the present study, we conducted a retrospective cohort study to explore the relationship between AIP levels and all-cause death and renal non-recovery, providing some reference for evaluation of short-term prognosis in diabetic patients with AKI.

## Methods

### Research subjects

Patients diagnosed with diabetes and AKI from April 2010 to April 2025 in the First Affiliated Hospital of Guangxi Medical University were retrospectively reviewed. Inclusion criteria: (1) diabetes was diagnosed before the occurrence of AKI; (2) changes in serum creatinine (Scr) consistent with the diagnostic criteria for AKI; (3) surviving patients had a follow-up period of no less than 90 days. Exclusion criteria: (1) age < 18 years; (2) CKD stage 5 or already undergoing regular renal replacement therapy (RRT); (3) patients with missing important baseline data such as renal function and lipid levels; (4) patients who discharged or died within 48h after admission. This study has been approved by the Ethics Committee of the First Affiliated Hospital of Guangxi Medical University [Approval Number: 2019(KY-E028)]. As this study is a retrospective analysis of anonymized clinical data and all patient identifiers have been removed, there is no need for patients to sign an informed consent form. We implemented strict protocols to ensure that all participant data has been anonymized and securely stored. This study was conducted in accordance with the tenets of the Declaration of Helsinki.

### Data collection

Demographic data and baseline clinical data were collected, including age, gender, smoking and alcohol drinking history, comorbidities (hypertension, cerebrovascular disease, CHD, congestive heart failure (CHF), CKD, infection and MODS), body mass index, blood pressure, laboratory parameters (blood routine, liver function, kidney function, blood glucose, blood lipid) and previous medications (including the use of contrast agents, lipid-lowering drugs, ACEI/ARB, SGLT2i). The baseline AIP level was calculated based on the formula AIP=log10 [TG (mg/dL)/HDL-C(mg/dL)]. All laboratory indicators were the first test values after hospitalization.

### Diagnostic criteria

AKI and stage were defined according to the diagnostic criteria of KDIGO guidelines (20): (1) diagnostic criteria of AKI: Scr increase ≥ 26.5μmol/L within 48h, or increase > 50% of baseline within 7 days. (2) The criteria of AKI stage: stage 1: Scr increase to 1.5–1.9 times of baseline value or increased ≥ 26.5μmol/L, stage 2: Scr increase to 2.0–2.9 times of base value, stage 3: Scr increase to 3 times or ≥ 353.6μmol/L or begin RRT. The diagnosis of diabetes was in line with the standards of the World (21): diabetes symptoms plus (1) random blood glucose ≥ 11.1mmol/L, or (2) fasting blood glucose (FBG) ≥ 7.0mmol/L, or (3) 2-hour blood glucose of oral glucose tolerance test ≥ 11.1mmol/L; patients who had been diagnosed with diabetes and were taking antidiabetic therapy. Renal function recovery was defined as a return of Scr to less than 1.25 times the baseline value or Scr decrease to normal lab range (Scr ≤ 104 mmol/L in males or Scr ≤ 84 mmol/L in females) or removal of RRT; Otherwise, it was defined as non-recovery of renal function (22). The baseline Scr was defined according to the following hierarchy: for patients with at least one Scr measurement recorded within the three months prior to hospital admission, the average value from that period was used. For patients lacking pre-admission Scr data, the average Scr level measured during hospitalization but before the onset of AKI was applied (23).

### Study grouping and clinical outcome

A retrospective cohort study was conducted. The follow-up time was no less than 90 days from the diagnosis of AKI. The primary endpoint was all-cause death, and the secondary endpoint was non-recovery of renal function at the last follow-up. According to the quartiles of AIP, the patients were divided into Q1 (AIP ≤ 0.66), Q2 (0.66 < AIP ≤ 0.88), Q3 (0.88 < AIP ≤ 1.10), and Q4 (AIP > 1.10) groups.

### Statistical analysis

SPSS22.0 and R4.5.2 were used for the statistical analysis. Quantitative data with a normal distribution are expressed as mean ± standard deviation, while non-normally distributed data are presented as median (interquartile range). Comparisons between groups were performed using analysis of variance for normally distributed data and non-parametric tests for non-normally distributed data. Categorical data are expressed as frequency (percentage), and group comparisons were made using the χ^2^ test. The Kaplan-Meier survival curve (Log-rank χ^2^ method) was used to compare the differences in survival rates of diabetic patients with AKI among different AIP groups. Receiver operating characteristic (ROC) curves were conducted to assess the predictive value of AIP for all-cause death and lack of renal recovery in diabetic patients with AKI. Cox regression analysis was used to identify the risk factors for a poor prognosis. Furthermore, we constructed and visualized restricted cubic spline (RCS) regressions (with 4 knots) to test the dose-response relationship between AIP level and all-cause death and lack of renal recovery. Stratified analyses were also performed to explore whether the association between AIP and prognosis varied by age, gender, smoking, comorbidities (CKD, MODS). Finally, a competing risk analysis was performed using the Fine-Gray model, treating death as a competing event for renal non-recovery.

## Results

### Baseline characteristics and clinical outcomes

As shown in **Table 1**, a total of 1046 diabetic patients with AKI were included in the analysis. There were 698 males (61.6%) and 348 females (38.4%), with a male-to-female ratio of 2:1. The average follow-up time was 101.13 ± 64.81 days. The all-cause mortality was 16.8% (176/1046). The main causes of death were infection in 54 cases (30.7%), cardiovascular and cerebrovascular diseases in 102 cases (58.0%), and other or unknown causes in 20 cases (11.4%). The rate of patients with renal non-recovery was 69.4% (604/870). The rate of RRT and using lipid-lowering drugs were 21.8% and 65.8%, respectively. The causes of AKI included nephrotoxins (353 cases, accounting for 33.7%), infection (327 cases, 31.3%), ischemic acute tubular necrosis (168 cases, accounting for 16.1%), and obstructive urinary tract diseases (198 cases, accounting for 18.9%). Among them, 728 cases (accounting for 69.6%) had stage 1 AKI, 124 cases (accounting for 11.9%) had stage 2 AKI, and 194 cases (accounting for 18.5%) had stage 3 AKI.

**Table 1:** Summary of baseline characteristics of the study population according to AIP quartile group.

Characteristic		AIP quartile				F/χ^2^/Z	P-value
	Overall	Q1 (AIP ≤ 0.66)	Q2 (0.66<AIP ≤ 0.88)	Q3 (0.88<AIP ≤ 1.10)	Q4 (AIP >1.10)		
No. of patients	1046	262	264	260	260		
Basic characteristics							
Gender (male), n (%)	644(61.6)	102(38.9)	200(75.8)	162(62.3)	180(69.2)	85.715	**<0.001**
Age, years	62.40 ± 13.57	62.55 ± 13.02	62.06 ± 13.02	63.30 ± 12.73	61.69 ± 15.37	0.681	0.564
Diabetes duration, month	43.56 ± 10.45	43.25 ± 10.98	43.01 ± 11.45	44.46 ± 12.76	45.16 ± 13.28	0.768	0.453
LOS, days	16(9,26)	16(10,25)	17(11,27)	18(10,28)	14(7,24)	16.586	**0.001**
Smoking, n (%)	390(37.3)	74(28.2)	104(39.4)	100(38.5)	112(43.1)	13.544	**0.004**
Alcohol drinking, n (%)	354(33.8)	56(21.4)	100(37.9)	100(38.5)	98(37.7)	24.312	**<0.001**
Contrast agent, n (%)	174(16.6)	36(13.7)	38(14.4)	56(21.5)	44(16.9)	7.063	0.070
ICU admission, n (%)	454(43.4)	102(38.9)	116(43.9)	120(46.2)	116(44.6)	3.120	0.373
RRT, n (%)	228(21.8)	50(19.1)	46(17.4)	78(30.0)	54(20.8)	14.517	**0.002**
Height, cm	163.03 ± 7.85	162.40 ± 7.60	164.04 ± 7.16	161.49 ± 8.48	164.18 ± 7.83	7.323	**<0.001**
Weight, kg	66.59 ± 33.01	62.88 ± 12.36	71.18 ± 61.98	64.30 ± 10.68	67.98 ± 13.62	3.401	**0.017**
BMI, kg/m^2^	25.02 ± 12.93	23.78 ± 4.07	26.52 ± 24.79	24.65 ± 3.72	25.12 ± 4.02	2.070	0.103
Comorbidities							
Hypertension, n (%)	612(58.5)	180(68.7)	104(39.4)	156(60.0)	172(66.2)	57.447	**<0.001**
Cerebrovascular disease, n (%)	268(25.6)	52(19.8)	70(26.5)	78(30.0)	68(26.2)	7.349	0.062
CHD, n (%)	234(22.4)	56(21.4)	64(24.2)	56(21.5)	58(22.3)	0.787	0.853
CHF, n(%)	354(33.8)	94(35.9)	88(33.3)	90(34.6)	82(31.5)	1.201	0.753
CKD, n (%)	208(19.9)	40(15.3)	58(22.0)	58(22.3)	52(20.0)	5.187	0.159
Infection, n (%)	218(20.8)	44(16.8)	38(14.4)	66(25.4)	70(26.9)	18.336	**<0.001**
MODS, n (%)	198(18.9)	36(13.7)	50(18.9)	58(22.3)	54(20.8)	7.104	0.069
AKI stage						29.256	**<0.001**
stage 1, n (%)	728(69.6)	174(66.4)	200(75.8)	156(60.0)	198(76.2)		
stage 2, n (%)	124(11.9)	34(13.0)	34(12.9)	38(14.6)	18(6.9)		
stage 3, n (%)	194(18.5)	54(20.6)	30(11.4)	66(25.4)	44(16.9)		
Laboratory parameters							
SBP, mmHg	133.97 ± 27.66	136.93 ± 25.19	137.52 ± 25.28	136.39 ± 30.66	124.99 ± 27.36	12.668	**<0.001**
DBP, mmHg	75.55 ± 16.82	77.34 ± 17.64	77.84 ± 14.67	76.38 ± 15.92	70.57 ± 17.93	10.731	**<0.001**
PP, mmHg	58.43 ± 19.41	59.59 ± 17.03	59.67 ± 18.49	60.01 ± 22.42	54.42 ± 18.89	5.010	**0.002**
MAP, mmHg	95.02 ± 18.97	97.21 ± 18.83	97.73 ± 16.75	96.38 ± 19.25	88.71 ± 19.61	13.470	**<0.001**
WBC, 10^12^/L	12.41 ± 6.59	11.68 ± 6.03	11.42 ± 4.50	12.79 ± 7.43	13.77 ± 7.70	7.155	**<0.001**
Hb, g/L	101.47 ± 25.98	104.6 ± 25.05	104.58 ± 26.02	100.48 ± 25.07	96.15 ± 26.93	6.383	**<0.001**
Platelets, 10^9^/L	205.05 ± 116.59	218.2 ± 98.65	215.36 ± 115.45	202.84 ± 115.62	183.51 ± 131.82	4.842	**0.002**
NEUT%	0.77 ± 0.15	0.75 ± 0.15	0.75 ± 0.15	0.78 ± 0.16	0.81 ± 0.13	8.849	**<0.001**
FBG, mmol/L	9.00 ± 4.05	7.72 ± 3.91	8.24 ± 3.35	9.37 ± 4.38	10.67 ± 3.88	29.558	**<0.001**
2h-PBG, mmol/L	13.40 ± 5.06	11.85 ± 4.66	12.95 ± 4.62	14.04 ± 5.31	14.76 ± 5.15	17.219	**<0.001**
HbA1c, %	8.66 ± 2.64	7.66 ± 2.15	8.25 ± 2.37	8.72 ± 2.52	10.03 ± 2.87	42.570	**<0.001**
ALB, g/L	31.34 ± 7.86	32.50 ± 7.73	32.62 ± 8.47	30.46 ± 7.84	29.74 ± 6.97	9.123	**<0.001**
BUN, mmol/L	15.41 ± 11.82	12.54 ± 9.16	14.39 ± 11.20	15.75 ± 12.30	19.00 ± 13.35	14.393	**<0.001**
Scr, μmol/L	134.55 ± 91.02	126.55 ± 88.93	134.67 ± 80.73	138.25 ± 104.92	138.79 ± 87.93	1.006	0.389
eGFR, mL/min per 1.73m^2^	73.92 ± 27.83	73.57 ± 28.85	74.48 ± 22.63	74.69 ± 33.16	72.93 ± 25.79	0.225	0.879
UA, μmol/L	413.41 ± 193.18	428.93 ± 175.11	412.71 ± 171.65	391.24 ± 202.95	420.2 ± 218.86	1.790	0.147
Cystatin C, mmol/L	2.49 ± 1.75	2.31 ± 1.52	2.09 ± 1.24	2.64 ± 1.90	2.93 ± 2.12	12.036	**<0.001**
TC, mg/dL	168.75 ± 93.11	166.69 ± 68.26	176.41 ± 68.33	170.21 ± 71.44	161.6 ± 142.55	1.172	0.319
Triglycerides, mg/dL	259.74 ± 203.19	118.78 ± 51.01	220.14 ± 122.65	318.33 ± 255.21	383.38 ± 207.51	111.543	**<0.001**
HDL-C, mg/dL	30.96 ± 14.81	40.78 ± 14.78	34.47 ± 13.08	29.21 ± 11.85	19.24 ± 9.97	137.615	**<0.001**
LDL-C, mg/dL	93.86 ± 53.23	99.01 ± 51.82	101.91 ± 52.32	99.09 ± 54.44	75.30 ± 50.15	14.762	**<0.001**
Medications							
Lipid-lowering drugs, n (%)	688(65.8)	167(63.7)	172(65.1)	176(67.6)	173(66.5)	0.963	0.810
ACEI or ARB, n (%)	180(17.2)	52(19.8)	42(15.9)	50(19.2)	36(13.8)	4.403	0.221
SGLT2i n (%)	319(30.5)	80(30.5)	81(30.7)	78(30.0)	80(30.8)	0.044	0.998
Clinical outcomes							
Death, n (%)	176(16.8)	0(0)	22(8.3)	48(18.5)	106(40.8)	173.610	**<0.001**
Non-recovery of renal function, n (%)	604(69.4)	108(41.2)	172(71.1)	190(89.6)	134(87)	161.678	**<0.001**

ACEI, angiotensin-converting enzyme inhibitor; AIP, atherogenic index of plasma; AKI, acute kidney injury; ALB, albumin; ARB, angiotensin II receptor blocker; BMI, body mass index; BUN, serum urea nitrogen; CHD, coronary heart disease; CHF, congestive heart failure; CKD, chronic kidney disease; DBP, diastolic blood pressure; eGFR, estimated glomerular filtration rate; FBG, fasting blood glucose; Hb hemoglobin; HbA1c, glycated hemoglobin; HDL-C, high-density lipoprotein cholesterol; ICU, intensive care unit; LDL-C, low-density lipoprotein cholesterol; LOS, length of stay; MAP, mean arterial pressure; MODS, multiple organ dysfunction syndrome; NEUT%, neutrophil percentage; PBG, postprandial blood glucose; PP, pulse pressure; RRT, renal replacement therapy; SBP, systolic blood pressure; Scr, serum creatinine; SGLT2, sodium-glucose co-transporter 2; TC, total cholesterol; UA, uric acid; and WBC, white blood cell.The boldfaced values indicate P < 0.05.

### Comparison of clinical data among different groups in quartiles of AIP

The average AIP of diabetic patients with AKI was 0.88 ± 0.34. Using the quartiles of AIP as cut-off values, there were 262 patients in the Q1 group (AIP ≤ 0.66), 264 patients in the Q2 group (0.66 < AIP ≤ 0.88), 260 patients in the Q3 group (0.88 < AIP ≤ 1.10), and 260 patients in the Q4 group (AIP > 1.10). As shown in **Supplementary Figure 1**, there was no statistically significant difference in the composition of AKI causes among the different groups in quartiles of AIP (χ^2^ = 6.219, *P* = 0.718).

Compared with the low AIP quartile group (Q1), the high AIP quartile group (Q4) of diabetic patients with AKI had higher infection incidence, white blood cell count (WBC), neutrophil percentage (NEU%), FBG, 2-hour post-meal blood glucose (2h-PBG), HbA1c, urea nitrogen (BUN), cystatin C, and TG levels, while hemoglobin, platelet count (PLT), albumin, and HDL-C levels were lower. Compared with the low AIP quartile group (Q1), the high AIP quartile group (Q4) of diabetic patients with AKI had higher rate of all-cause death and renal non-recovery (*P* < 0.05).

### Kaplan-Meier survival curves for the all-cause death in different AIP quartiles

**Figure 1:**
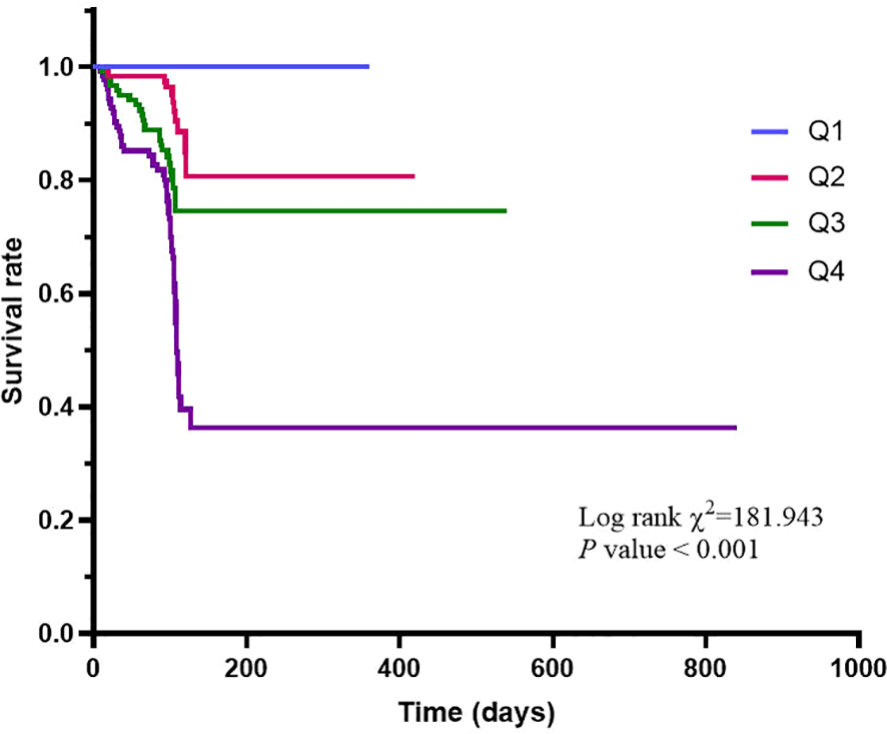
Kaplan-Meier survival curves for the all-cause death comparing diabetic patients with AKI in different AIP quartiles.

As shown in **Figure 1**, the Kaplan-Meier survival analysis showed that the average survival time of the AIP quartile groups were Q1 group [107.11 days (95% CI = 100.92-113.30)], Q2 group [105.28 days (95% CI = 98.06-112.51)], Q3 group [96.80 days (95% CI = 88.75-104.85)], and Q4 group [95.24 days (95% CI = 85.57-104.91)], respectively. The cumulative survival rate of the Q4 group was significantly lower than that of the Q1, Q2, and Q3 groups (100% vs 80.6% vs 74.6% vs 36.3%; Log-rank χ^2^ = 181.943, *P* < 0.001), and the all-cause mortality also increased with the increase of the AIP quartile group.

### The predictive effect of AIP on poor prognosis

**Figure 2:**
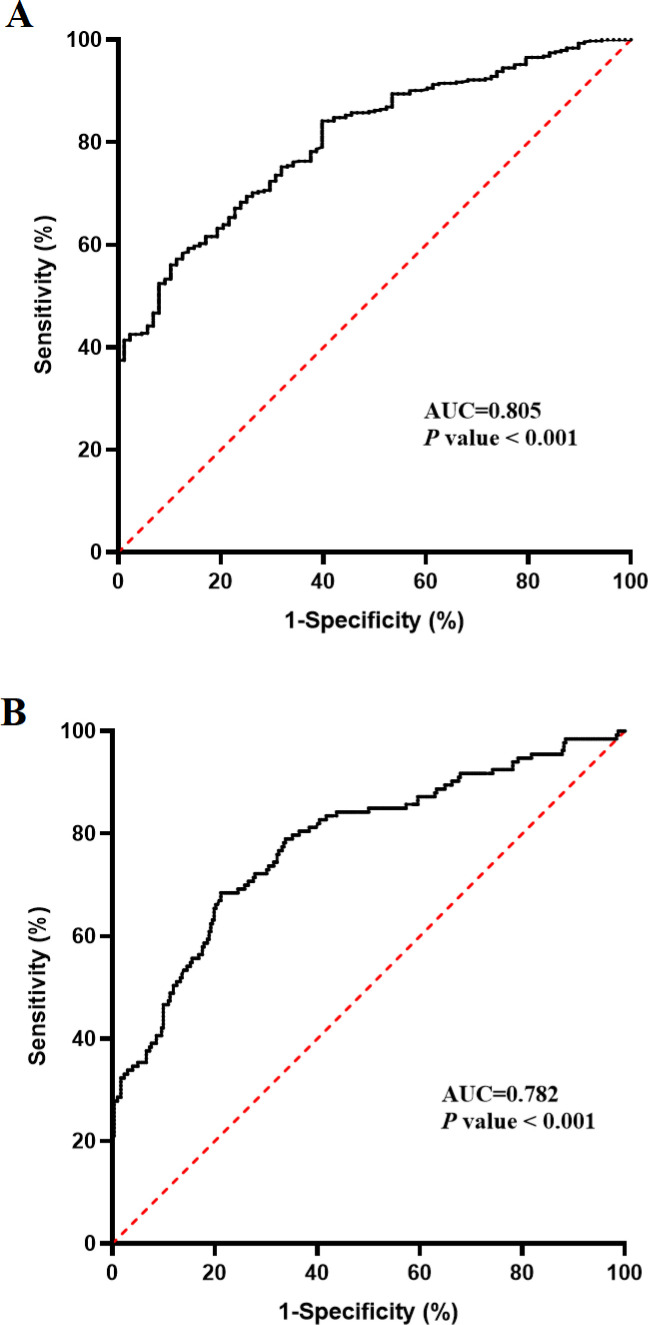
The ROC curve of AIP for predicting all-cause death **(A)** and non-recovery of renal function **(B)**.

The ROC curve analysis showed that the area under the curve for AIP in predicting all-cause death in diabetic patients with AKI was 0.805 (95% CI: 0.774-0.835), and the optimal cutoff value of AIP was 0.861. At this point, the sensitivity was 0.898 and the specificity was 0.739, with a Youden index of 0.637 (**Figure 2A**). The area under the curve for AIP in predicting the non-recovery of renal function in diabetic patients with AKI was 0.782 (95% CI = 0.747-0.817), and the optimal cutoff value of AIP was 0.708. At this point, the sensitivity was 0.788 and the specificity was 0.784, with a Youden index of 0.572 (**Figure 2B**).

### Risk factors of all-cause death

As shown in **Table 2**, univariate Cox regression analysis indicated that age, smoking, infection, MODS, mean arterial pressure, WBC, hemoglobin, PLT, NEU%, FBG, 2h-PBG, HbA1c, BUN, and AIP were associated with all-cause death in diabetic patients with AKI. Variables with *P* < 0.05 were included in the multivariate Cox regression analysis. All the variables in the model satisfied the proportional hazard (PH) assumption, and there was no significant multicollinearity (the variance inflation factor values were less than 5, as shown in **Supplementary Table 1**). The results showed that age (hazard ratio (HR)=1.031, 95%CI=1.018-1.044), smoking (HR = 2.595, 95%CI=1.859-3.622), MODS (HR = 2.561, 95%CI=1.726-3.799), and WBC (HR = 1.062, 95%CI=1.035-1.189), hemoglobin (HR = 0.989, 95%CI=0.982-0.996), PLT (HR = 0.998, 95%CI=0.996-0.999), HbA1c (HR = 1.400, 95%CI=1.288-1.521) and AIP (HR = 5.427, 95%CI=2.999-9.821) were independent risk factors for all-cause death in diabetic patients with AKI. When AIP was categorized into quartiles (Q1–Q4) and entered into the multivariate Cox model with Q2 as the reference, the adjusted HRs for all-cause death in diabetic patients with AKI were 2.719 (95% CI = 1.572-4.703) for Q3, and 2.932 (95% CI = 1.753-4.902) for Q4 (P for trend < 0.001).

**Table 2:** Cox regression analysis of all-cause mortality in diabetic patients with AKI.

Characteristic	Univariate analysis			Multivariate analysis		
	HR	HR 95%CI	P-value	HR	HR 95%CI	P-value
Age, years	1.021	1.010-1.032	<0.001	1.031	1.018-1.044	**<0.001**
Smoking	2.356	1.751-3.169	<0.001	2.595	1.859-3.622	**<0.001**
Infection	1.767	1.283-2.435	<0.001	1.463	0.842-2.541	0.177
MODS	2.360	1.676-3.324	<0.001	2.561	1.726-3.799	**<0.001**
MAP, mmHg	0.976	0.968-0.984	<0.001	1.006	0.996-1.016	0.230
WBC, 10^12^/L	1.037	1.018-1.157	<0.001	1.062	1.035-1.189	**0.007**
Hb, g/L	0.972	0.966-0.978	<0.001	0.989	0.982-0.996	**0.002**
Platelets, 10^9^/L	0.994	0.992-0.996	<0.001	0.998	0.996-0.999	**0.005**
NEUT%	68.811	17.176-275.665	<0.001	3.719	0.917-15.084	0.066
FBG, mmol/L	1.175	1.151-1.199	<0.001	1.050	0.992-1.111	0.092
2h-PBG, mmol/L	1.106	1.087-1.125	<0.001	0.983	0.934-1.034	0.506
HbA1c, %	1.447	1.387-1.509	<0.001	1.400	1.288-1.521	**<0.001**
ALB, g/L	0.945	0.927-0.963	<0.001	0.999	0.973-1.026	0.941
BUN, mmol/L	1.002	1.001-1.003	<0.001	1.010	0.997-1.022	0.124
AIP (continuous, HR per 1-unit increase)	18.817	11.980-29.556	<0.001	5.427	2.999-9.821	**<0.001**
AIP (quartile)						
Q2	1 (Reference)			1 (Reference)		
Q3	2.630	1.587-4.357	<0.001	2.719	1.572-4.703	**<0.001**
Q4	5.969	3.769-9.454	<0.001	2.932	1.753-4.902	**<0.001**

AIP, atherogenic index of plasma; ALB, albumin; BUN, serum urea nitrogen; CHD, coronary heart disease; FBG, fasting blood glucose; Hb hemoglobin; HbA1c, glycated hemoglobin; MAP, mean arterial pressure; MODS, multiple organ dysfunction syndrome; NEUT%, neutrophil percentage; PBG, postprandial blood glucose; and WBC, white blood cell. The multivariate model for all-cause death was adjusted for the following covariates: age (continuous), smoking (yes/no), MODS (yes/no), WBC (continuous), hemoglobin (continuous), platelet count (continuous), HbA1c (continuous), and AIP (continuous/categorical variable). The boldfaced values indicate P < 0.05.

### Risk factors of non-recovery of renal function in survivors

As shown in **Table 3**, the univariate Cox regression analysis indicated that smoking, drinking, CKD, MODS, FBG, 2h-PBG, HbA1c, TC, LDL-C, and AIP were associated with the non-recovery of renal function in diabetic patients with AKI. Variables with *P* < 0.05 were included in the multivariate Cox regression analysis. All the variables in the model satisfied the PH assumption, and there was no significant multicollinearity (as shown in **Supplementary Table 2**). The results showed that smoking (HR = 1.305, 95% CI = 1.044-1.633), CKD (HR = 1.321, 95% CI = 1.077-1.620), MODS (HR = 1.517, 95% CI = 1.222-1.883), FBG (HR = 1.059, 95% CI = 1.030-1.089), and AIP (HR = 2.769, 95% CI = 2.085-3.677) were independent risk factors for the non-recovery of renal function in surviving diabetic patients with AKI. When AIP was categorized into quartiles and entered into the multivariate Cox model with Q1 as the reference, the adjusted HRs for non-recovery of in diabetic patients with AKI who survived were 1.817 (95% CI = 1.422-2.322) for Q2, 2.509 (95% CI = 1.966-3.203) for Q3, and 2.218 (95% CI = 1.690-2.909) for Q4 (P for trend < 0.001).

**Table 3:** Cox regression analysis of non-recovery of renal function in diabetic patients with AKI.

Characteristic	Univariate analysis			Multivariate analysis		
	HR	HR 95%CI	P-value	HR	HR 95%CI	P-value
Smoking	1.405	1.191-1.657	<0.001	1.305	1.044-1.633	**0.020**
Alcohol Drinking	1.405	1.190-1.659	<0.001	1.189	0.952-1.486	0.127
CKD	1.323	1.085-1.613	0.006	1.321	1.077-1.620	**0.007**
MODS	1.665	1.359-2.042	<0.001	1.517	1.222-1.883	**<0.001**
FBG, mmol/L	1.059	1.039-1.079	<0.001	1.059	1.030-1.089	**<0.001**
2h-PBG, mmol/L	1.030	1.015-1.046	<0.001	0.995	0.973-1.018	0.681
HbA1c, %	1.046	1.011-1.082	0.009	0.983	0.941-1.026	0.422
TC, mg/dL	1.002	1.001-1.003	0.001	1.001	0.999-1.003	0.191
LDL-C, mg/dL	1.002	1.000-1.003	0.029	1.002	0.999-1.004	0.227
AIP (continuous, HR per 1-unit increase)	2.788	2.161-3.596	<0.001	2.769	2.085-3.677	**<0.001**
AIP (quartile)						
Q1	1 (Reference)			1 (Reference)		
Q2	1.823	1.432-2.320	<0.001	1.817	1.422-2.322	**<0.001**
Q3	2.497	1.971-3.164	<0.001	2.509	1.966-3.203	**<0.001**
Q4	2.306	1.787-2.976	<0.001	2.218	1.690-2.909	**<0.001**

AIP, atherogenic index of plasma; CKD, chronic kidney disease; FBG, fasting blood glucose; HbA1c, glycated hemoglobin; LDL-C, low-density lipoprotein cholesterol; MODS, multiple organ dysfunction syndrome; PBG, postprandial blood glucose; and TC, total cholesterol. The multivariate model for non-recovery of renal function was adjusted for the following covariates: smoking (yes/no), drinking (yes/no), CKD (yes/no), MODS (yes/no), FBG(continuous), and AIP (continuous/categorical variable). The boldfaced values indicate P < 0.05.

### Nonlinear analysis of AIP and clinical outcomes

**Figure 3:**
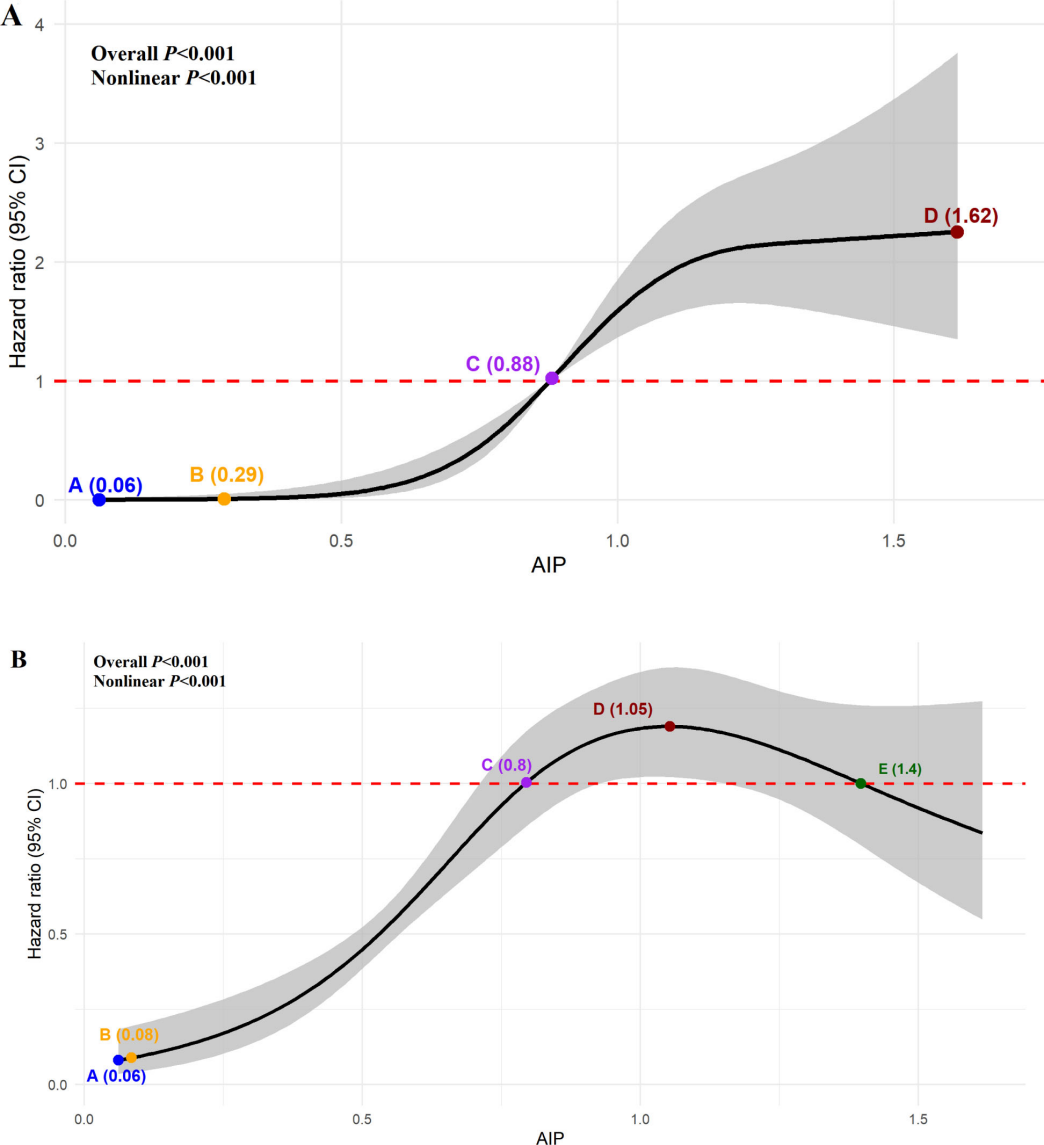
Dose-response curves of AIP and all-cause mortality **(A)** and non-recovery of renal function **(B)**. The solid black line represents the HR, and the gray shaded area indicates the 95% CI. The horizontal red dashed line indicates HR = 1 (reference). In **(A)**, the minimum AIP value (0.06) was used as the reference. Points A-D indicate key thresholds: A (AIP = 0.06, strongest protection), B (AIP = 0.29, steepest increase), C (AIP = 0.87, protective-to-hazard transition), and D (AIP = 1.62, highest risk); the model was adjusted for age (continuous), smoking (yes/no), MODS (yes/no), WBC (continuous), hemoglobin (continuous), platelet count (continuous), and HbA1c (continuous). In **(B)**, the minimum AIP value (0.06) was used as the reference. Points A–E indicate key thresholds: A (AIP = 0.06, left protection), B (AIP = 0.08, steepest increase), C (AIP = 0.80, protective-to-risk transition), D (AIP = 1.05, peak risk), and E (AIP = 1.40, risk-to-protective transition); the model was adjusted for smoking (yes/no), CKD (yes/no), and FBG (continuous).

The RCS regression analysis revealed a significant S-shaped non-linear association between AIP and all-cause death (**Figure 3A**, P for nonlinear <0.001). As AIP increased from 0.06 to 1.62, the risk of all-cause death demonstrated a complete transition from strong protection (HR = 0.0008) to significant hazard (HR = 2.250). Based on the curve morphology, the relationship between AIP and all-cause death was categorized into a slow increase phase (AIP 0.06-0.29), a rapid increase phase (AIP 0.29-0.87), and another slow increase phase (AIP 0.87-1.62). The steepest increase in risk of all-cause death occurred at AIP = 0.29, the transition from protection to hazard occurred at AIP = 0.87, and the highest risk was observed at AIP = 1.62.

In addition, the RCS regression analysis revealed a significant inverted U-shaped non-linear association between AIP and non-recovery of renal function (**Figure 3B**, P for nonlinear < 0.001). The risk of renal function non-recovery initially increased with rising AIP, reached a peak, and then declined. According to the curve shape, the relationship between AIP and non-recovery of renal function was divided into left protective zone (AIP < 0.80): lower AIP levels were associated with reduced risk; intermediate risk zone (AIP 0.80-1.40): AIP levels in this range were associated with increased risk, with the highest risk observed at AIP = 1.05; right protective zone (AIP > 1.40): higher AIP levels were again associated with reduced risk.

### Subgroup analysis

As shown in **Table 4**, the subgroup analyses showed that MODS significantly modified the effect of AIP on both clinical outcomes in diabetic patients with AKI (all-cause death: P for interaction=0.009; non-recovery of renal function: P for interaction=0.017), with stronger associations observed in patients with MODS. No significant interactions were found for age, sex, or smoking (all P for interaction > 0.05).

**Table 4:** Subgroup analysis of the role and differences of AIP in prognosis of diabetic patients with AKI.

Characteristic	All-cause death		Non-recovery of renal function	
	HR	HR 95%CI	HR	HR 95%CI
Age, years				
18–59	4.802	2.106-10.951	3.140	2.137-4.615
≥60	5.342	1.860-15.343	2.193	1.350-3.561
P-interaction	0.736		0.154	
Sex				
Male	3.061	1.498-6.256	2.934	2.080-4.139
Female	9.326	2.240-38.825	2.929	1.720-4.986
P-interaction	0.577		0.748	
Smoking				
Yes	4.416	1.861-10.479	1.894	1.123-3.196
No	14.692	5.383-40.102	3.363	2.350-4.812
P-interaction	0.244		0.664	
CKD				
Yes	3.298	2.860-5.891	5.907	2.381-14.655
No	4.360	2.310-8.226	2.544	1.868-3.466
P-interaction	0.050		**0.027**	
MODS				
Yes	9.131	2.312-36.055	4.808	2.114-10.931
No	4.259	2.101-8.633	3.029	2.216-4.141
P-interaction	**0.009**		**0.017**	

CKD, chronic kidney disease; MODS, multiple organ dysfunction syndrome. The boldfaced values indicate P < 0.05.

### Sensitivity analysis

**Figure 4:**
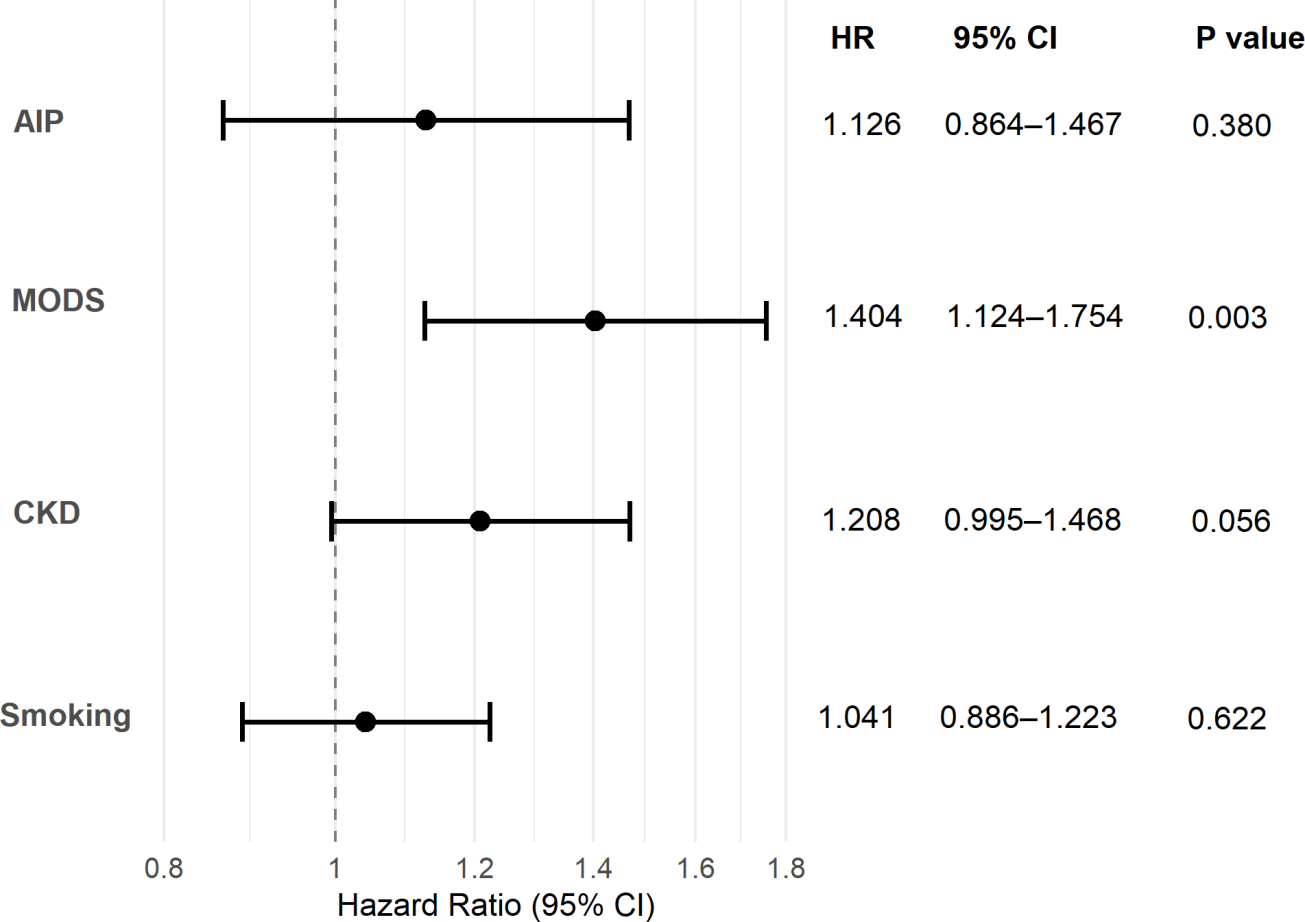
Sensitivity analysis: Fine-Gray competing risk model for non-recovery of renal function. AIP, atherogenic index of plasma; CKD, chronic kidney disease; MODS, multiple organ dysfunction syndrome.

In the sensitivity analysis using the Fine-Gray model (**Figure 4**), after accounting for all-cause death as a competing event, AIP was no longer significantly associated with non-recovery of renal function in diabetic patients with AKI (n=1046, HR = 1.126, 95% CI: 0.864–1.467, P = 0.380).

## Discussion

This present study explored the relationship between AIP levels and the poor prognosis of diabetic patients with AKI through a retrospective cohort study. Cox regression showed that after adjusting for multiple confounding factors, the level of AIP remained a risk factor for all-cause death (HR = 5.427, 95%CI=2.999-9.821) and non-recovery of renal function (HR = 2.769, 95% CI = 2.085-3.677) in diabetic patients with AKI. Notably, RCS analyses revealed distinct nonlinear patterns for the two outcomes: an S-shaped curve for all-cause death and an inverted U-shaped curve for renal non-recovery. These findings suggest that the prognostic value of AIP for diabetic patients with AKI extends beyond simple linear association, reflecting complex dose-response relationships. The identification of key inflection points of AIP for prognosis provides clinically relevant targets for risk stratification and intervention. To our knowledge, this is the first study to investigate the relationship between AIP and clinical outcomes of diabetic patients with AKI.

Our study found that cardiovascular and cerebrovascular diseases were the leading cause of death among diabetic patients with AKI (accounting for 58.0%). Studies indicated that atherosclerosis was the principal macrovascular complication of diabetes and a primary etiology of cardiovascular disease (CVD), with dyslipidemia being a key driver of its pathogenesis (24, 25). AIP is a valuable integrated marker for assessing lipid metabolic disorders and predicting atherosclerosis risk. A change of 1 unit in AIP usually reflects a significant change in the metabolic status. As a novel and practical lipid parameter, AIP quantifies the capacity for glucose and lipid metabolism, thereby serving as a valuable tool for assessing risk of atherosclerosis (26, 27). Previous studies demonstrated that AIP could be directly used for the risk assessment of diabetes and prediabetes, and its clinical application in metabolic disorders associated with diabetes had great potential (28–30). Our study identified that the level of AIP was associated with all-cause death in diabetic patients with AKI. In a nationwide cohort study (31), the risk of diabetes mortality rose significantly across increasing AIP quartiles. Compared to Q1, the adjusted HRs for Q2, Q3, and Q4 were 1.22 (0.52-2.84), 1.28 (0.60-2.73), and 2.86 (1.38-5.94), respectively (*P* = 0.001), indicating a significant association between AIP and diabetes mortality after multivariable adjustment, which was consistent with our research. The association between AIP and all-cause death in diabetic patients with AKI may stem from potential mechanisms involving atherosclerosis and insulin resistance (IR). AIP reflects the ratio of TG to HDL-C. Elevated TG levels promote the formation of small and dense LDL-C particles, which can increase the risk of atherosclerosis progression and occurrence through effects such as promoting vascular endothelial damage, oxidative stress, and lipid deposition (32, 33). Thereby increasing the risk of cardiovascular and cerebrovascular diseases and affecting all-cause mortality. In addition, patients with diabetes often have IR, which is related to the increase in AIP levels (34, 35). IR may contribute to vascular endothelial dysfunction by reducing the utilization of nitric oxide (NO), activating the sympathetic nervous system, stimulating the proliferation and migration of vascular smooth muscle cells. These effects accelerate atherosclerosis, promote vasoconstriction and elevated blood pressure, and consequently increase the risk of CVD (36, 37), and ultimately increasing the all-cause mortality. AIP may affect the all-cause death of diabetic patients with AKI through the above mechanisms. However, these mechanisms explain only the overall positive association between AIP and all-cause death, but not the differential effects across its range. RCS analysis in this study revealed a significant S-shaped nonlinear relationship, suggesting a threshold effect in AIP. The harmful impact of AIP becomes evident only after exceeding 0.29, and beyond 0.87, the risk of all-cause death continues to increase but at a decelerating rate. This S-shaped relationship reflects the dynamic interaction between lipotoxicity and compensatory capacity (38). When AIP levels are low, the body’s compensatory mechanisms effectively maintain homeostasis; however, once AIP exceeds a certain threshold, lipotoxicity becomes the predominant factor (39), accompanied by a rapid increase in the risk of cardiovascular events and all-cause death. The specific pathophysiological process still needs to be further clarified through basic research.

Our research results showed that the rate of renal non-recovery in surviving diabetic AKI patients is as high as 69.4%, which is similar to previous study (66.8%) (40). This high rate likely reflects the combined impact of impaired renal repair mechanisms in diabetes, the high proportion of severe AKI (stage 3: 18.5%) and MODS (18.9%), and the high prevalence of baseline CKD (19.9%) in our cohort. In addition, previous studies showed that AIP was associated with the risk and prognosis of AKI in patients with pancreatitis (41), critically ill (42), and sepsis (43). Our results also showed that level of AIP was associated with renal non-recovery in diabetic patients with AKI who survived. Diabetes and AKI share overlapping pathophysiological pathways, including oxidative stress, inflammation, and endothelial dysfunction. These pathways are further exacerbated by dyslipidemia, which enhances susceptibility to AKI and impedes renal recovery (44, 45). Under hyperglycemic and ischemic conditions, excess TG accumulate in renal cells as lipid droplets, resulting in ectopic lipid deposition, promoting tubular apoptosis and lipotoxicity-related renal injury (46). Moreover, normal HDL also contributes to maintaining endothelial function and NO production, which is essential for tissue perfusion and prevention of leukocyte adhesion and infiltration (47). When AIP increases, the deficiency and/or dysfunction of HDL can lead to severe infiltration of inflammatory cells in renal tissue, which may exacerbate the severity of oxidative stress and inflammation, and promote the progression of renal arteriosclerosis (48). Elevated AIP may cause persistent renal damage through the above mechanisms, which is related to the renal prognosis of diabetic patients with AKI. However, RCS analysis revealed a significant inverted U-shaped relationship between AIP and renal non-recovery (P for nonlinearity < 0.01). AIP levels of 0.80–1.40 were associated with increased risk, while risk paradoxically declined when AIP exceeded 1.40. This apparent biphasic effect is explained by competing risk from death (49). Renal outcome analysis included only 870 survivors (176 deaths excluded). Patients with extremely high AIP (>1.40) had the highest mortality risk (as shown in **Figure 3A**), and thus were excluded from renal prognosis analysis. The remaining high-AIP patients represent a selected survivor population, artificially lowering the observed risk of renal prognosis. Competing risk analysis confirmed this interpretation: after accounting for death as a competing event, the association between AIP and renal non-recovery was no longer significant (HR = 1.126, P = 0.380), in contrast to standard Cox regression (HR = 2.769, P<0.001). Thus, AIP is primarily a predictor of mortality; its apparent association with renal outcomes is largely driven by competing mortality risk.

Notably, our study showed that MODS was the only factor that significantly modified the association between AIP and both clinical outcomes (all-cause death: P = 0.009; non-recovery of renal function: P = 0.017). This indicates that diabetic AKI patients with MODS constitute a high-risk subgroup particularly susceptible to AIP-related adverse outcomes. The systemic inflammatory state induced by MODS and the lipotoxicity reflected by AIP may exert a synergistic amplification effect (50, 51), collectively contributing to an unfavorable prognosis. In clinical practice, these such high-risk patients should be prioritized for intensive lipid monitoring and comprehensive management strategies.

This study has several limitations. First, it was a single-center retrospective study with a relatively limited sample size and short follow-up duration, which can only assess the correlation between AIP and prognosis, rather than a causal relationship. Second, potential confounding factors that might influence AIP and outcomes in diabetic patients with AKI (antibiotics, vasopressor, and mechanical ventilation) were not included in the analysis. Third, the diagnosis of AKI was based solely on changes in Scr without incorporating urine output criteria, possibly missing patients diagnosed exclusively by oliguria. Finally, reliance on a single baseline AIP measurement may underestimate the true impact of AIP dynamics on clinical outcomes. Therefore, future studies should incorporate longitudinal AIP monitoring and involve multicenter, large-sample cohorts to further elucidate the pathophysiological mechanisms of AIP in diabetic patients with AKI.

## Conclusion

In conclusion, AIP is an independent risk factor for all-cause death in diabetic patients with AKI, showing an S-shaped nonlinear relationship, with a stronger effect in those with MODS. The observed association between AIP and non-recovery of renal function is substantially influenced by competing mortality risk rather than a direct causal relationship. AIP may serve as a useful marker for mortality risk stratification, while its relationship with renal outcomes should be further investigated.

## Data Availability

The raw data supporting the conclusions of this article will be made available by the authors, without undue reservation.
