# Supplementary material for: Atherogenic index of plasma is associated with poor prognosis in diabetic patients with acute kidney injury
Source: Front Endocrinol (Lausanne). 2026 Apr 16;17:1796454. doi: 10.3389/fendo.2026.1796454 (PMC13128647; doi:10.3389/fendo.2026.1796454)
Supplement: Supplementary file 1 [file DataSheet1.docx]

**Table S1.** Collinearity diagnostics between AIP and other covariates when all-cause death was the dependent variable.

| **Variable** | **VIF** |
| --- | --- |
| Age | 1.052 |
| Smoking | 1.041 |
| MODS | 1.025 |
| WBC | 1.040 |
| Hb | 1.057 |
| Platelets | 1.038 |
| HbA1c | 1.151 |
| AIP | 1.159 |

**Abbreviation**: AIP, atherogenic index of plasma; Hb hemoglobin; HbA1c, glycated hemoglobin; MODS, multiple organ dysfunction syndrome; VIF, variance inflation factor; and WBC, white blood cell. all VIF values were below 5, indicating no substantial multicollinearity.

**Table S2.** Collinearity diagnostics between AIP and other covariates when non-recovery of renal function was the dependent variable.

| **Variable** | **VIF** |
| --- | --- |
| Smoking | 1.807 |
| Drinking | 1.814 |
| CKD | 1.006 |
| MODS | 1.012 |
| FBG | 1.807 |
| AIP | 1.814 |

**Abbreviation**: AIP, atherogenic index of plasma; CKD, chronic kidney disease; FBG, fasting blood glucose; MODS, multiple organ dysfunction syndrome; PBG, postprandial blood glucose; and VIF, variance inflation factor. all VIF values were below 5, indicating no substantial multicollinearity.


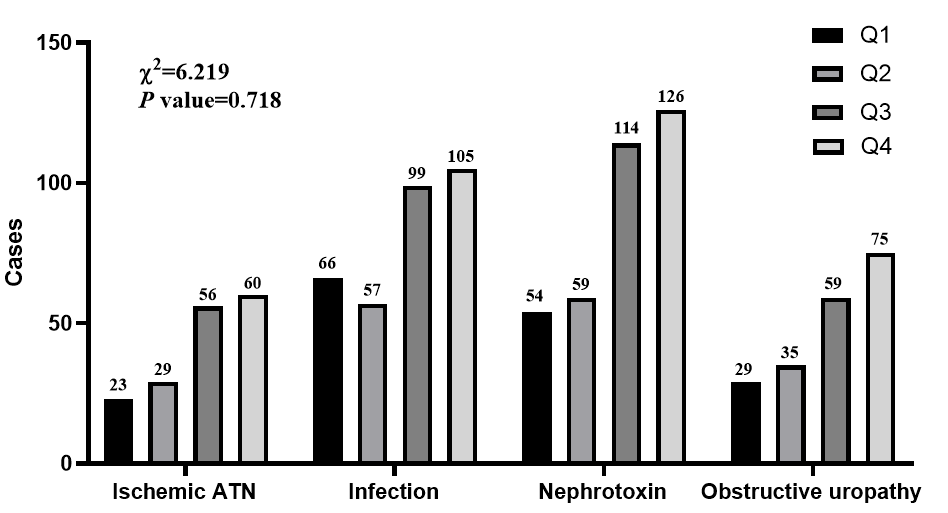


Figure S1. The distribution of AIP quartiles in different groups according to AKI etiology
